# Standardization of Methods for Early Diagnosis and On-Site Treatment of High-Altitude Pulmonary Edema

**DOI:** 10.1155/2011/190648

**Published:** 2011-06-01

**Authors:** Qiquan Zhou

**Affiliations:** Department of High Altitude Disease, College of High Altitude Military Medicine, Third Military Medical University, and Key Laboratory of High Altitude Medicine, Ministry of Education, Chongqing 400038, China

## Abstract

High-altitude pulmonary edema (HAPE) is a life-threatening disease of high altitude that often affects nonacclimatized apparently healthy individuals who rapidly ascend to high altitude. Early detection, early diagnosis, and early treatment are essential to maintain the safety of people who ascend to high altitude, such as construction workers and tourists. In this paper, I discuss various methods and criteria that can be used for the early diagnosis and prediction of HAPE. I also discuss the preventive strategies and options for on-site treatment. My objective is to improve the understanding of HAPE and to highlight the need for prevention, early diagnosis, and early treatment of HAPE to improve the safety of individuals ascending to high altitude.

## 1. Introduction

High-altitude pulmonary edema (HAPE) is a specific disease of high altitude. It has a high incidence and is often serious because of its rapid progresses. It is also life-threatening if treatment is not started in a timely manner [1, 2].

Many studies [3, 4] have shown that HAPE is a major disease that often affects nonacclimatized healthy individuals who ascend to high altitudes. Therefore, early diagnosis of HAPE is essential to initiate early treatment of HAPE and maintain the safety of people who ascent to high altitude. Although international and Chinese criteria have been established for HAPE [5], its early detection and diagnosis are difficult, except in the presence of typical pulmonary edema. Following the magnitude 7.1 earthquake that struck Yushu (Yushu Tibetan Autonomous Prefecture, Qinghai Province, China) on April 14, 2010, approximately 50,000 rescuers assembled in the region. This region lies at an altitude ranging from 3700 m to 4900 m, the average altitude is 4,493 m. Although the incidence of HAPE is only 2–4%, the sudden influx of nonacclimatized rescuers to the region meant that HAPE was one of the most common life-threatening diseases at that time, even considering the effects of the earthquake, which caused 1944 deaths, with 216 people missing and 12,135 injured.

HAPE can also induce psychological disorders in affected individuals. Therefore, it is essential to highlight the early diagnosis and on-site treatment of HAPE. Under the support of the National Science and Technology Program, we have conducted an extensive range of studies on HAPE intended to find effective methods for early diagnosis and clinical treatment of HAPE and, thus, improve the health and safety of individuals who rapidly ascend to high altitude.

## 2. Methods for the Early Diagnosis of HAPE

It is generally not difficult for physicians to diagnose typical HAPE, which is based on medical history, symptoms, and signs including white, yellow, or pink frothy sputum, moist rales on pulmonary auscultation, and flocculent shadows on chest X-rays. However, atypical HAPE is much more difficult to diagnose. Patients usually develop atypical HAPE at altitude <3000 m, which may occur several days or longer after ascending to altitude. Symptoms differ from those of typical HAPE, such as less sputum or an absence of sputum and nonspecific findings on chest X-rays. Based on a previous study [6], the development of HAPE can be classified into two stages from the onset to the presence of typical symptoms. The early stage is characterized by interstitial pulmonary edema while the late stage is characterized by alveolar pulmonary edema. Patients with interstitial pulmonary edema do not usually exhibit serious dyspnea or typical signs such as pink frothy sputum, extensive moist rales, or wheezy phlegm. Thus, these patients are susceptible to misdiagnosis and delayed treatment. Li et al. [7] investigated the characteristics of HAPE in 482 patients. They examined the clinical symptoms and signs, and performed routine blood tests, electrocardiography, color Doppler echocardiography, chest X-rays, and computed tomography (CT) of the patients. They found that HAPE patients exhibited significant differences in these parameters compared with non-HAPE patients, and that the symptoms and signs were more obvious in HAPE patients than in non-HAPE patients. In addition, studies have revealed that oxygen treatment is more effective in HAPE than in other forms of lung injury. Therefore, some researchers have proposed that tentative oxygen treatment can be used for the early diagnosis of HAPE. Nevertheless, the most important examinations for the early diagnosis of HAPE are chest X-rays or CT. On chest X-rays, HAPE often presents as decreased pulmonary transmittance, increased or obscure lung markings, and ground glass-like changes in the lung, or patchy shadows. On CT scans, increased and enlarged lung markings, ground glass-like changes in the lung, nodule-like shadows, scattered or isolated alveolar edema of terminal bronchioles, and slim reticulate shadows can be observed.

## 3. Diagnosis and Grading of HAPE

### 3.1. Criteria for Early Diagnosis of HAPE

According to the clinical symptoms, signs, and findings of blood routine tests, electrocardiography, color Doppler echocardiography, chest X-rays, and CT, the following criteria for HAPE have been developed [7]:

1. recent ascent to high altitude (>3000 m); the presence of palpitations, chest tightness, dyspnea and cough with or without white foamy sputum;
2. local, unilateral, or bilateral coarse breath sounds, with or without local moist rales, central cyanosis, tachycardia (>100/min), and tachypnea (>24/min);
3. chest X-ray findings including decreased pulmonary transmittance, increased or obscure lung markings, ground glass-like changes or patchy shadows in the lung; CT findings including increased and enlarged lung markings, ground glass-like changes, nodule-like shadows, scattered or isolated alveolar edema of terminal bronchioles and slim reticulate shadows;
4. routine blood test findings include an increased white blood cell count and an increased neutrophil count;
5. arterial blood gas analysis showing continuous hypoxemia accompanied by mild respiratory alkalosis;
6. electrocardiographic findings including sinus tachycardia, clockwise rotation and sharp P waves;
7. persistent pulmonary hypertension on echocardiography;
8. symptoms resolve rapidly following rest, oxygen treatment, pulmonary artery pressure lowering treatment, and diuresis.

For the diagnosis of HAPE, criteria 1, 2 and 3 must be met. These findings, together with those in 4, 5, 6, 7, and/or 8, are then used to confirm HAPE. Using these criteria, the severity of disease can be determined, which is critical for individualized therapy. With early diagnosis and early treatment, HAPE can be controlled at the early stage and the symptoms be markedly improved.

### 3.2. Criteria for the Grading of HAPE

Once HAPE is diagnosed, its severity should be graded to provide individualized therapy. Therefore, when a patient with suspected HAPE is admitted, the grade of HAPE should be confirmed as soon as possible according to the criteria for early diagnosis [8]. 

#### 3.2.1. Mild

Dyspnea and cough with white foamy sputum may occur after intermediate manual labor. Lung auscultation shows local moist rales in a unilateral lung. The respiratory rate is often <24 breaths/min, and heart rate <100 beats/min. There is no arrhythmia. Chest X-rays shows the area of flocculent shadows occupies <1/4 of the lung. The shadows are confined to the right lower lobe and are spotty or patchy. CT scans reveal increased and enlarged lung markings. Routine blood tests are normal.

#### 3.2.2. Moderate

Dyspnea, chest pain, chest tightness, and cough with a large amount of white foamy sputum occur after mild manual labor. Extensive moist rales are noted in the bilateral lower lung or unilateral lung on lung auscultation. The respiratory rate is >24 breaths/min while the heart rate is >110 beats/min and is accompanied by arrhythmia. Chest X-rays show patchy or flocculent shadows covering >1/2 of the lung, and CT scans show ground glass-like changes or nodule-like shadows. The white blood cell count and neutrophil count are slightly increased.

#### 3.2.3. Severe

Patients can not lie in the supine or prone position. They may have a pale complexion, cold sweat on the forehead, serious dyspnea, and a heavy cough with a large amount of white or pink foamy sputum. Rales of small, intermediate, and large bubbles are extensive in the bilateral lungs and are accompanied by the sound of boiling water. The respiratory rate is >30 breaths/min, while the heart rate is >120 beats/min and is accompanied by arrhythmia. Chest X-rays reveal asymmetric cloudy shadows covering >1/2 of the bilateral lungs, and CT scans indicate scattered or isolated alveolar edema of the terminal bronchioles. The white blood cell count is 10 × 10^9^/L.

#### 3.2.4. Extremely Severe

The symptoms and signs are more serious than those in severe HAPE. Patients are at high risk of dying. They have a pale complexion, weak breathing, and a large amount of foam is discharged from the nose and mouth. A gurgling sound is audible in the bilateral lungs. The heart sounds are weak, and blood pressure is decreased. The patient is also likely to have high-altitude cerebral edema. Heart failure and secondary pulmonary infection are very likely. Chest X-rays reveal flocculent shadows in unilateral or bilateral lungs, enlargement of the heart, and the pulmonary artery is particularly clear. CT scans show diffuse alveolar edema in unilateral or bilateral lungs. The number of white blood cells is >16 × 10^9^/L.

## 4. Guidelines for On-Site Treatment of HAPE

### 4.1. Guidelines for the Treatment of HAPE

In terms of pharmacotherapy, there is no consensus on the types of drugs, their doses, or administration routes for HAPE. Thus, it is imperative to develop guidelines to standardize and optimize the treatment of HAPE. Based on the experience of several mountain sickness treatment centers and the efficacy of currently available drugs and strategies, in combination with recent advances in the treatment of HAPE, our research group proposed four regimens for early diagnosis and treatment of HAPE and conducted a prospective, randomized, controlled study to investigate their efficacy. A total of 400 patients with HAPE were divided into four groups and treated as follows: patients in group A received oxygen inhalation, dexamethasone, and aminophylline; patients in group B-C were treated as in group A, in addition to diuresis with furosemide in group B, blood pressure-lowering with nifedipine in group C, or L-arginine in group D. The efficacy and safety of these therapeutic regimens were compared. The treatment showing greatest therapeutic efficacy was subsequently used to prepare the guidelines for the treatment of HAPE. Overall, all four regimens were effective for the treatment of HAPE [9]. However, in terms of the time to resolution of symptom and signs, the time to resolution of chest X-ray findings, and duration of hospitalization, regimen B was superior to the other regimens. Interestingly, there were no marked differences between regimens A, C, and D. Furthermore, there were no differences in the incidence of adverse effects between regimens A, B, and D, or in the liver and kidney functions between all four regimens. These findings suggest that a regimen composed of oxygen inhalation, dexamethasone, aminophylline, and furosemide is effective, well tolerated, simple to follow, and thus offers a basic, standardized regimen for the treatment of HAPE 

Once the regimen has been selected, timely initiation of individualized treatment is a key factor that contributes towards its therapeutic efficacy. Our experience of on-site treatment of more than 300 patients with HAPE has shown that on-site individualized treatment is feasible, without increasing the risk for death [10]. Following the Yushu earthquake, the efficacy of this regimen was further confirmed as the prognosis after on-site treatment was significantly better than that after blind evacuation, which usually results in poor outcomes.

### 4.2. Principles of Individualized Treatment of HAPE

The principles of individualized treatment of HAPE are highly dependent on the severity of HAPE [11].

#### 4.2.1. Mild

Bed rest, intermittent oxygen inhalation or subcutaneous oxygen treatment, oral aminophylline (250 mg), prednisone (10 mg), furosemide (20 mg), atropine or anisodamine (5 mg) twice daily, and other treatments for specific symptoms are recommended.

#### 4.2.2. Moderate

Absolute bed rest, continuous oxygen inhalation or subcutaneous oxygen treatment are necessary. Drugs are mainly administered intramuscularly, accompanied by oral and intravenous medication. Aminophylline (250 mg), prednisone (10 mg), furosemide (20 mg), atropine or anisodamine (5 mg) three to four times daily, and other treatments for specific symptoms are recommended.

#### 4.2.3. Severe

Absolute bed rest, preferably in a semirecumbent position and continuous high-flow oxygen inhalation with defoaming agents in a humidifier bottle are necessary. Drugs are mainly administered intravenously and intramuscularly. Atropine (2–5 mg/0.5 h) or anisodamine (20–40 mg/0.5 h), dexamethasone (10 mg/4 h), furosemide (40 mg/8 h), gentamicin (80000 IU/8 h); or dexamethasone (200 mg), furosemide (40 mg), atropine or anisodamine (10 mg), and gentamicin (160000 IU) in 10% glucose solution (500 mL) three times daily are recommended. Symptomatic treatments may include intramuscular morphine (10 mg) for patients with dysphoria, intramuscular cedilanid (0.4–0.8 mg) for patients with heart failure, and vitamin C, ATP solution, coenzyme A, and cytochrome C, as deemed necessary.

#### 4.2.4. Extremely Severe

On-site treatment is preferred, followed by escalation therapy during evacuation once the patient's condition has stabilized. The procedures for the treatment of extremely severe HAPE are similar to those of severe HAPE, except for the treatment of complications. For patients with cerebral edema, the doses of dexamethasone and diuretics should be increased and airway maintenance is necessary. Hyperbaric oxygen treatment or oxygen inhalation through a ventilator is also recommended. Hypothermia is beneficial for the brain. For patients with serious heart failure, half or two-thirds of the recommended dose of digitalis should be administered in combination with an appropriate dose of a sedative. The daily dose of glucose should be <400 g to maintain low energy consumption and to promote osmotic diuresis. The treatments should be escalated during evacuation once the patient's condition has stabilized. 

The following factors are particularly important during evacuation. (1) Mode of transport: the patient should be transported in a vehicle that can provide rapid and steady evacuation. Such transportation options include helicopter, motor truck, heavy-duty medical car, and miniambulance. The vehicle's speed should be carefully controlled, particularly in undulating conditions, to ensure the patient remains in a horizontal position and excess movement is avoided. (2) Accompanying personnel: the patient should be ideally accompanied by one nurse and one physician, or at least one medical staff who can provide effective on-site treatment, The personnel should continuously monitor and record the patient's vital signs and perform treatment as deemed necessary. (3) Patient position: the patient should be kept in a semirecumbent position, and airway maintenance is essential. However, the supine position is recommended for patients in a coma. Head vibration and bumps should be avoided to prevent cerebral hernia. (4) For deceased patients, the time of death and the patients symptoms and signs at death should be recorded, and the body should remain in a stable position.

#### 4.2.5. Symptomatic Treatment

For patients with suspected heart failure, treatment with digitalis (e.g., cedilanid and digoxin) is recommended to improve myocardial contractility. For patients with low blood pressure, dextran and hydroxyethyl starch 40 is recommended for blood volume expansion. For patients with secondary infections, penicillin or other antibiotics can be administered. For patients with dysphoria, sedatives can be given according to the disease state. 

In summary, patients with mild to moderate HAPE should receive basic treatment plus any supplementary treatment required. Patients with severe and extremely severe HAPE should also receive treatment for any symptoms. Basic treatment consists of bed rest, oxygen inhalation, and intravenous administration of aminophylline, dexamethasone, and furosemide. Supplementary treatments include antibiotics, vitamin C, and cytochrome C, for example.

### 4.3. Pathophysiological Evidence for the On-Site Treatment of HAPE

#### 4.3.1. Bed Rest

Physical activity may lead to contraction of pulmonary vessels resulting in an increase in pulmonary artery pressure and a decrease in arterial partial pressure of oxygen. Therefore, absolute bed rest can reduce muscular activity and contraction of blood vessels and, thus, provide stable pulmonary artery pressure. In some studies [12], bed rest alone was used for the treatment of mild HAPE.

#### 4.3.2. Oxygen Inhalation

Oxygen inhalation can decrease the pulmonary artery pressure. Evidence shows that inhalation of 100% oxygen can significantly decrease the increased pulmonary artery pressure and is accompanied by improvements in symptoms [13]. In addition, oxygen inhalation can increase arterial oxygen saturation and improve tissue oxygen supply, thus alleviating hypoxia. For patients with severe HAPE, hyperbaric oxygen is preferred. Some researchers [14, 15] have proposed mechanical ventilation for patients with respiratory distress. Hyperbaric oxygen treatment not only increases the oxygen concentration and subsequently the alveolar and arterial partial pressure of oxygen, but also increases pulmonary ventilation and alveolar pressure, which inhibit fluid exudation. Oxygen inhalation can also rectify respiratory alkalosis, which prevents the transportation of peripheral blood to the lung.

#### 4.3.3. Nitric Oxide Inhalation

Experimental studies have shown that hypoxia results in reduced nitric oxide levels, a principle cause of pulmonary vasoconstriction and that inhalation of nitric oxide can reduce hypoxic pulmonary hypertension, improve the patient's ventilation to perfusion ratio, improve hypoxemia, and improve HAPE in patients with signs and symptoms, as well as decrease hospitalization time with few adverse effects. Accordingly, we suggest that nitric oxide inhalation should be considered in the treatment of HAPE. It should be administered via a nasal catheter at a dose of 10 ppm (0.001%) at a flow rate of 3–5 L/min for 30 min in normal air or in oxygen.

#### 4.3.4. Aminophylline

Aminophylline is a classic bronchodilator and has long been used in the treatment of HAPE. Aminophylline can dilate the bronchus, decrease hypoxic pulmonary hypertension, enhance diaphragm function, suppress lipid peroxidation and hypoxia-induced pulmonary vascular inflammation, enhance cardiac function and diuresis, clear bronchial mucus, and subsequently resolve moist rales. Unlike other bronchodilators, aminophylline can dilate both the bronchus and vascular smooth muscle; hence, it is preferred over other bronchodilators for the treatment of HAPE.

#### 4.3.5. Anticholinergics

Some hospitals use anisodamine (654-2) instead of aminophylline for the treatment of HAPE. Anisodamine can improve pulmonary vascular spasms, decrease pulmonary vascular resistance and hypoxic pulmonary hypertension, improve the pulmonary microcirculation, and maintain smooth pulmonary blood flow. As a result, anisodamine inhibits intravascular coagulation and, thus, reduces the risk of pulmonary embolism.

#### 4.3.6. Nifedipine

Nifedipine is a calcium channel blocker that inhibits release of catecholamines from sympathetic nerve endings. Therefore, it is an effective vasodilator, reduces hypoxic pulmonary hypertension, increases arterial oxygen tension, improves the symptoms and signs of HAPE, lowers right atrial pressure, and increases cardiac output. Therefore, nifedipine targets many of the effects of HAPE.

#### 4.3.7. Dehydration and Diuresis

Hypoxia can lead to redistribution of systemic blood and abrupt increases in pulmonary blood volume. Furosemide and chlorothiazide not only promote diuresis and dehydration, but also increase renal blood flow and reduce left ventricular filling pressure. Thus, blood in the lungs is transported to the periphery, improving pulmonary congestion. In addition, oral acetazolamide can play a significant role in the treatment of HAPE because it can increase urine output in patients with HAPE or fluid retention. Acetazolamide is a carbonic anhydrase inhibitor that inhibits carbonic anhydrase activity in the brain, kidney, and blood. In the kidney, acetazolamide reduces the formation of hydrogen and bicarbonate ions and inhibits sodium bicarbonate reabsorption to increase urine output and, thus, reduce sodium and water retention. In the brain, acetazolamide inhibits brain choroid plexus carbonic anhydrase activity to reduce cerebrospinal fluid production rate and hence reduce intracranial pressure.

#### 4.3.8. Dexamethasone

Dexamethasone is an analog of adrenocortical hormones that can improve the functions of capillary endothelial cells and alveolar epithelial cells, decrease pulmonary capillary permeability, protect alveolar epithelial type II cells, promote the secretion of pulmonary surfactant, increase renal blood flow, and decrease the secretion of antidiuretic hormone. Thus, dexamethasone is a key drug in the treatment of HAPE.

## 5. Early Prediction of HAPE

HAPE is a disease that is very difficult to predict. Although much research has been done in attempts to predict HAPE, no consensus has been reached regarding markers for the early prediction of HAPE. However, many studies have suggested that HAPE could be predicted based on physiological functions, molecular biology, or genetic predisposition.

### 5.1. Physiological Parameters

#### 5.1.1. Oxygen Saturation

Respiratory gas exchange disorders may predict serious hypoxemia and even acute mountain sickness in individuals who rapidly ascend to high altitude. Thus, monitoring arterial oxygen saturation (SaO_2_) can be used to predict HAPE. Studies have shown that 80–100% of patients with serious hypoxemia develop HAPE. Accordingly, Roach et al. [16] speculated that noninvasive measurement of SaO_2_ was a simple and convenient way to predict the onset of HAPE. Similarly, Burtscher et al. [17] suggested that measurement of SaO_2_ could be used to predict the onset of acute mountain sickness. In a prospective cohort study, Shen et al. [18] found that there were significant individual differences in the changes in oxygen saturation with increases in altitude. They found that when the SaO_2_ decreased by >30% at low altitude, the susceptibility to HAPE increased markedly and about 62% of the participants developed HAPE. Thus, they speculated that a decrease in SaO_2_ > 30% offers a biomarker to predict susceptibility to HAPE.

#### 5.1.2. Maximal Oxygen Uptake

Zhang et al. [19] used submaximal bench stepping to directly calculate maximal oxygen uptake (VO_2max_), which was then applied to predict HAPE. They conducted this study in soldiers ascending to high altitude. They found that soldiers with a VO_2max_ ≥ 3 L/min at sea level were less likely to develop HAPE than those with a VO_2max_ < 3 L/min.

#### 5.1.3. Forced Vital Capacity (FVC)

Wang and Zhou [20] measured FVC, body surface area, and thoracic volume in an effort to predict HAPE in individuals ascending to high altitude. They found that a FVC/body surface area <3 L/m^3^, FVC/thoracic volume <400 L/m^3^, lung/body index >30, and lung/thorax index >0.24 were associated with increased risk for HAPE.

#### 5.1.4. Pulmonary Diffusion Function

Geng et al. [21] investigated the diffusion of carbon monoxide (CO) in the lung of 27 subjects who rapidly ascended from 2260 m to 4700 m. They found that CO diffusion was correlated with the development of acute mountain sickness. In 10 patients with HAPE, the diffusing capacity of CO (DLCO) increased with increasing altitude, but the amplitude of this increase was greater in subjects without HAPE. Thus, they speculated that insufficient DLCO was a cause of acute mountain sickness and that DLCO is an objective parameter to predict adaptability to high altitude and the occurrence of HAPE.

#### 5.1.5. Urine Volume after a Water Load

Huang et al. [22] measured urine volume within 2.5 h after participants administered 1000 mL of water at sea level in an effort to predict HAPE. They found that urine volume in subjects who developed acute mountain response was significantly lower than that in the control group. In addition, the urine volumes at 1, 1.5, 2, and 2.5 h after hydration and the total urine volume within 1.5, 2, and 2.5 h after hydration were negatively associated with AMS. They concluded that greater urine volume within 2.5 h after hydration at sea level was associated with milder symptoms of acute mountain sickness after ascending to high altitude. Therefore, urine volume after hydration might be a marker for acute mountain sickness.

#### 5.1.6. Blood Pressure and Blood pH

Zhou et al. [23] measured the pH of blood in individuals before they ascended to high altitude, in addition to PaCO_2_, and PaO_2_. In that study, the blood pH was significantly associated with susceptibility to HAPE, and there was a significant difference in blood pH value between subjects who developed HAPE and without HAPE. They found that during intense physical labor following a rapid ascent to 4000 m, the likelihood of developing acute mountain sickness was significantly greater among individuals with blood pH > 7.45 than among those with blood pH < 7.45. However, they found no significant differences in PaCO_2_ or PaO_2_ between these two groups of subjects. Thus, they concluded that blood pH, but not PaCO_2_ or PaO_2_, could be used as a predictor of HAPE.

#### 5.1.7. Ventilatory Function

Zhou et al. [24] investigated the ventilatory functions in 113 newly recruited soldiers at sea level, and then the soldiers were transferred to 3658 m by airplane. Two and three days after ascending to high altitude, the investigators scored the symptoms of acute mountain sickness and evaluated the relationship between ventilatory functions and symptom scores. They found that FVC, peak expiratory flow, and forced expiratory volume in 1 second in soldiers with HAPE were significantly lower than those in soldiers without HAPE. Thus, they speculated that ventilatory functions at sea level could be used to screen subjects susceptible to HAPE.

#### 5.1.8. Cardiopulmonary Exercise Test

Wu and Li [25] investigated whether a cardiopulmonary exercise test could be used to predict HAPE. They found that subjects with poor cardiac and ventilatory responses to hypoxia were more susceptible to HAPE. They assessed several parameters, including the heart rate to oxygen saturation ratio (ΔHR/ΔSaO_2_) and the minute ventilation to oxygen saturation ratio (ΔVE/ΔSaO_2_), to establish a mathematical model that could predict HAPE. They established a discriminant model based on *Z* = *b*_1_ *X*_1_ + *b*_2_ *X*_2_, in which coefficients *b*_1_ = 0.00769, *b*_2_ = 0.0810, and *Z*_0_ = 0.472 (discrimination threshold). In this model, *Z* > *Z*_0_ is defined as non-AMS and *Z* < *Z*_0_ is defined as AMS. The discriminant model had a high sensitivity (93.9%) and a high accuracy (92.2%) for the prediction of HAPE.

### 5.2. Endocrine Parameters

Endocrine parameters may also be used to predict HAPE. For example, Cai and Yang [26] used radioimmunoassays to measure the blood levels of 18 endocrine parameters, including thyroid stimulating hormone, adrenocorticotropic hormone, and corticotropin hormone, and found that plasma cortisol <20 *μ*g/L and urine 17-hydroxy corticosteroid (17-OHCS) levels <17 *μ*moL/dL could predict HAPE with an accuracy >95%.

### 5.3. Molecular Biological Parameters

Luo et al. [27–29] compared the sequences of the mitochondrial genome in HAPE patients and rats at high altitude versus sequences from healthy individuals at high altitude and animals at sea level, and identified single-nucleotide polymorphisms in nine genes in mitochondrial DNA (T6680C, C3970T, G3010A, A13497G, c15508T, G4164G, G1598A, C16111T, and T7684C). All of the affected genes encoded proteins controlling mitochondrial electron transport. Based on single-nucleotide polymorphism of mitochondrial DNA T6680C, DNA C3970T, and DNA G3010A, they developed a kit that can be used to predict susceptibility to HAPE. This kit can be used to screen subjects for susceptibility to HAPE before ascending to high altitude.

In another study, Qiu et al. [30] compared genetic parameters of patients with HAPE, as well as people living in Tibetan and Han Chinese migrants. They found that human leucocyte antigen (HLA) DR6, particularly DR6(1402), was associated with increased susceptibility to HAPE. Thus, they speculated that susceptibility to HAPE was not only related to genetic factors but was also influenced by some susceptibility genes.

## 6. Early Prevention of HAPE

Prevention of HAPE, as well as its associated symptoms and signs, is important to reduce its incidence among those ascending to high altitude. To achieve this, the following factors are particularly important. (1) Maintain body warmth with appropriate insulation and avoid upper respiratory tract infection. Several days before ascending to high altitude, bathing should be avoided to reduce exposure to cold temperatures. (2) Avoid fatigue. Physical activity at high altitude should be performed at an intensity of no more than 60–80% of that at sea level and duration of activity should be <6 h. Similarly, the body must be given sufficient time to rest both before and during the time at high altitude. (3) Stop alcohol intake. Fifteen days before ascending to high altitude, the consumption of alcohol should be stopped. (4) Emotional stress. Unwanted emotional tension should be avoided, while optimism and aggressiveness should be maintained. (5) Patients with upper respiratory tract infection should be carefully monitored before and during the time at high altitude. However, delaying the ascent until the infection has been fully resolved is preferable. (6) Oxygen must be available at high altitude, either by maintaining high oxygen levels in living quarters or by supplying compressed oxygen tanks for use during physical activity. (7) Food and water sanitation. Diets containing high levels of carbohydrates and protein and low levels of fat are recommended. The intake of vitamins can also be increased. (9) The use of drugs such as *Rhodiola rosea*, Zangtianlu, Codonopsis compound tablets, Shenqi pollen tablets, and acetazolamide during the time at high altitude can also reduce the risk of HAPE.

For people working at high altitude, such as construction workers, preventative measures are particularly important. As outlined above, it is essential to provide sufficient nutrients, rest, and insulation against the cold, as well as to limit labor intensity and duration of labor to avoid fatigue. Shift-work is also recommended and may entail 20–30 days of work at high altitude followed by a period of rest at low altitude to provide more effective resting conditions. Shift-work also reduces the incidence of chronic mountain sickness induced by long periods of physically demanding work at high altitude. It is also essential to ensure there is an adequate medical team to enable early detection, diagnosis, and treatment, should mountain sickness of HAPE occur.

Finally, individuals planning on ascending to high altitude should receive education regarding the prevention and treatment of HAPE and take appropriate measures [31] to decrease the risk of developing HAPE: maintain a positive attitude after ascending to high altitude take measures to prevent HAPE, and consider the possible treatments be optimistic but do not fear high altitude maintain body temperature avoid hyperphagia stop smoking and alcohol consumption conduct appropriate excise but avoid strenuous exercise and fatigue do not worry about trouble sleeping but try to maintain good sleep quality pay attention to cough, particularly cough event of bloody sputum and consider seeking treatment consider bed rest and oxygen inhalation be aware that headache, vomiting, and unsteady walking are symptoms of HAPE and should be checked by a physician; treatment should be started as soon as possible, even if the symptoms are mild, as timely and comprehensive treatment favors positive outcomes.

## References

[1] Hu ZQ (1963). High altitude pulmonary edema. *National Medical Journal of China*.

[2] Singh T (1965). High altitude pulmonary edema. *Lancet*.

[3] Basnyat B, Subedi D, Sleggs J (2000). Disoriented and ataxic pilgrims: an epidemiological study of acute mountain sickness and high altitude cerebral edema at a sacred lake at 4300 m in the Nepal Himalayas. *Wilderness & Environmental Medicine*.

[4] Ren Y, Fu Z, Shen W (2010). Incidence of high altitude illnesses among unacclimatized persons who acutely ascended to Tibet. *High Altitude Medicine & Biology*.

[5] Wu TY (1995). Evaluation of criteria for the diagnosis of high altitude diseases. *Journal of High Altitude Medicine*.

[6] Zhou QQ, Luo YJ, Li H (2010). Epidemiological study of mountain sickness complicated with multiple organ dysfunction syndrome on the Qinghai-Tibetan Plateau: report of 103 cases. *Scientific Research and Essays*.

[7] Li SZ, Wang YL, Yan CC (2010). Diagnostic criteria for high altitude pulmonary edema in the early stage. *Military Medical Journal of South China*.

[8] Yang JY, Wei WY, Wang XP (1986). Grading and on-site treatment of acute high altitude pulmonary edema: experience from 54 patients. *Chinese Journal of Tuberculosis and Respiratory Diseases*.

[9] Li SZ, Zheng BH, Yan CC (2010). Four therapeutic regimens for high altitude pulmonary edema: result comparison and preliminary study of standardized treatment. *Medical Journal of National Defending Forces in Southwest China*.

[10] Yang JY, Wang FS (1993). On-site treatment of acute high altitude deseases. *People’s Military Surgeon*.

[11] Zhang DM, Zhou QQ, Yang JY (2001). A clinical analysis of 306 cases acute high altitude pulmonary edema treated on the spot in high altitude regions. *West China Medical Journal*.

[12] Zafren K, Reeves JT, Schoene R (1996). Treatment of high-altitude pulmonary edema by bed rest and supplemental oxygen. *Wilderness & Environmental Medicine*.

[13] Xinbing M, Suzhi L, Yuqi G (2003). Haemodynamic changes in high altitude pulmonary edema and effects of oxygen breathing. *Chinese Journal of Pathophysiology*.

[14] Yuanda X, Mei J, Zheng lun X (2010). Noninvasive ventilation for treatment of acute respiratory failure secondary to severe acute respiratory syndrome. *Chinese Journal of Respiratory and Critical Care Medicine*.

[15] Ma SQ, Wu SZ, Yang ZP (2010). Treatment of high altitude pulmonary edema combined with secondary adult respiratory distress syndrome and monitoring of oxygen dynamics. *Medical Journal of National Defending Forces in Northwest China*.

[16] Roach RC, Greene ER, Schoene RB, Hackett PH (1998). Arterial oxygen saturation for prediction of acute mountain sickness. *Aviation Space and Environmental Medicine*.

[17] Burtscher M, Flatz M, Faulhaber M (2004). Prediction of susceptibility to acute mountain sickness by SaO_2_ values during short-term exposure to hypoxia. *High Altitude Medicine & Biology*.

[18] Shen Q, Sun YJ, Qi Y (2009). Magnitude of SaO_2_ decreasing with increasing altitude as a biomarker to predict HAPE occurred at high altitude. *Journal of Medical Research*.

[19] Zhang XZ, Zhang SP, Zhou ZJ (1996). Application of maximal oxygen uptake at sea level in the prediction of acute mountain sickness: an experimental study. *Medical Journal of National Defending Forces in Northwest China*.

[20] Wang LA, Zhou QQ (2003). Predictive importance of forced vital capacity to susceptive population of acute mountain sickness. *West China Medical Journal*.

[21] Geng D, Ge RL, Wang ZG (1995). Measurement of pulmonary diffusion capacity as a method evaluating acute mountain response in men exposed rapidly to high altitude. *Journal of High Altitude Medicine*.

[22] Huang QY, Gao YQ, Liu FY (2003). Correlation between the amount of urine excreted after water lod at sea level and the symptomatic scores of AMS. *Journal of High Altitude Medicine*.

[23] Zhou B, Zhou QQ, Yang JY (2004). Prediction of blood acid-base scale to the susceptible population with acute mountain sickness. *West China Medical Journal*.

[24] Zhou QQ, Gao YQ, Huang QY (2004). Predictive effect of lung functional determination of the population susceptible to acute mountain sickness. *Medical Journal of National Defending Forces in Northwest China*.

[25] Wu TY, Li WS (1992). Cardiopulmonary exercise test for the evaluation of acute mountain sickness. *Chinese Journal of Applied Physiology*.

[26] Cai WC, Yang JY (1990). Role of 18 endocrine parameters in the prediction of acute mountain sickness in susceptible population. *Ningxia Medical Journal*.

[27] Luo Y, Gao W, Gao Y (2008). Mitochondrial genome analysis of Ochotona curzoniae and implication of cytochrome c oxidase in hypoxic adaptation. *Mitochondrion*.

[28] Luo Y, Tang S, Gao W (2010). Genotyping mitochondrial DNA single nucleotide polymorphisms by PCR ligase detection reactions. *Clinical Chemistry and Laboratory Medicine*.

[29] Gao W, Gao Y, Zhang G, Song L, Sun B, Shi J (2005). Hypoxia-induced expression of HIF-1alpha and its target genes in umbilical venous endothelial cells of Tibetans and immigrant Han. *Comparative Biochemistry and Physiology Part C*.

[30] Qiu CC, Zhu TC, Sun YJ (2005). Molecular genetic study of different susceptibility to high altitude pulmonary edema. *Chinese Journal of Applied Physiology*.

[31] Zhou QQ (2010). How to practice scientific remedy in high altitude—about medical rescue after intense earthquake in Yushu Qinghai. *Chinese Journal of New Drugs*.

